# Supplementary material for: Sympathetic innervation of interscapular brown adipose tissue is not a predominant mediator of OT-elicited reductions of body weight gain and adiposity in male diet-induced obese rats
Source: bioRxiv. 2024 Sep 19:2024.09.12.612710. Preprint. [Version 2] doi: 10.1101/2024.09.12.612710 (PMC11430106; doi:10.1101/2024.09.12.612710)
Supplement: Supplementary file 1 [file NIHPP2024.09.12.612710v2-supplement-1.pdf]

bioRxiv preprint doi: https://doi.org/10.1101/2024.09.12.612710; this version posted September 19, 2024. The copyright holder for this preprint (which was not certified by peer review) is the author/funder. This article is a US Government work. It is not subject to copyright under 17 USC 105 and is also made available for use under a CC0 license.

Supplemental Study 1: Determine if surgical denervation of IBAT blocked the ability of 4V OT to increase T_{IBAT} in lean rats. The goal of this study was to determine if OT- elicited increase in T_{IBAT} requires intact SNS outflow to IBAT in lean rats. By design, rats were lean as determined by both BW (511.6 ± 2.4 g) and adiposity (3.3 ± 0.3 g fat mass; 18.1 ± 11.1% adiposity) after maintenance on the chow (13% kcal from fat; N=6/group) for approximately 1 week prior to denervation procedures and implantation of temperature transponders underneath IBAT. Rats were otherwise treated identically to those used in **Study 4**.

Supplemental Study 1:

Tissue samples from **Supplemental Study 1** were screened for NE content and compared with a previous cohort of sham-operated rats from **Study 1**. However, there was no age-matched cohort to use for comparison purposes to calculate reduction of IBAT NE content as the cohort used in **Supplemental Study 1** was heavier than the sham-operated cohort used in **Study 1** (354 ± 4.6 g) [(F(1,9) = 608.553, *P*<0.001]. IBAT NE content from sham-operated animals was only used to screen whether IBAT NE content was above the acceptable threshold for a successful IBAT denervation. NE content was reduced by 93.8 ±2.6% in IBAT from denervated rats relative to IBAT from sham-operated control rats [(F(1,9) = 16.390, *P*<0.01]. IWAT NE content was also reduced in the denervated group [(F(1,9) = 7.411, *P*<0.05)] but this might have been due, in part, to the differences in age of the animals between cohorts. In contrast, NE content was unchanged in pancreas but elevated in liver [(F(1,9) = 8.684, *P*<0.05] in denervated rats relative to sham rats.

In denervated rats, 4V OT (5 μg/μL) increased T_{IBAT} at 0.5, 0.75, 1, 1.25, 1.5 and 1.75-h post-injection (*P*<0.05; **Supplemental Figure 1A**). The high dose (5 μg/μL) also stimulated T_{IBAT} when measuring change in T_{IBAT} relative to baseline T_{IBAT} at 0.5, 0.75, 1, 1.25, 1.5, 1.75 and 2-h post-injection (*P*<0.05; **Supplemental Figure 1B**). The low dose (1 μg/μL) increased T_{IBAT} at 1.5-h post-injection (*P*<0.05; **Supplemental Figure 1A**). The low dose (1 μg/μL) tended to stimulate T_{IBAT} when measuring change in T_{IBAT} relative to baseline T_{IBAT} at 1- and 1.5-h post-injection (0.05<*P*<0.1; **Supplemental Figure 1B**).

bioRxiv preprint doi: https://doi.org/10.1101/2024.09.12.612710; this version posted September 19, 2024. The copyright holder for this preprint (which was not certified by peer review) is the author/funder. This article is a US Government work. It is not subject to copyright under 17 USC 105 and is also made available for use under a CC0 license.

Supplemental Study 2: Determine the extent to which β3-AR-induced activation of IBAT requires activation of β3-AR to increase T_{IBAT} in DIO rats.

Rats were fed *ad libitum* and maintained on HFD for approximately 4.5 months prior to receiving 4V cannulas and/or temperature transponders [28]. Rats were allowed to recover for at least 4 weeks during which time they were adapted to a daily 4-h fast, handling and mock injections.

The goal of this study was to determine a dose of the β3-AR antagonist that was sufficient to block the effects of the β3-AR agonist on T_{IBAT} in DIO rats. The dose of the β3-AR antagonist would then be used in **Study 6** to determine if 4V OT required β3-AR signaling to stimulate T_{IBAT} in DIO rats. By design, DIO rats were obese as determined by both BW (712 ± 23.1 g) and adiposity (217 ± 12.1 g fat mass; 31 ± 1.0% adiposity) after maintenance on the HFD for 4.5 months prior to sham/denervation procedures.

In the absence of the β3-AR antagonist, the β3-AR agonist increased T_{IBAT} at 0.25, 0.5, 0.75, 1, 1.5, 0.75, 2 and 4-h post-injection (P<0.05) and tended to stimulate T_{IBAT} at 180-min post-injection (0.05<P<0.1) (**Supplemental Figure 2**). These effects were blocked at 0.5-h post-injection of the β3-AR agonist. Together, these findings confirm the specificity of a β3-AR mediated effect of the β3-AR agonist on T_{IBAT} and identified a dose of the β3-AR antagonist that was sufficient to block the effects of the β3-AR agonist to stimulate T_{IBAT}.

Supplemental Study 3: Determine the extent to which OT-induced activation of sympathetic outflow to IBAT contributes to its ability to elicit weight loss in rats with more pronounced diet-induced obesity.

Rats were fed *ad libitum* and maintained on HFD for approximately 8.75 months prior to receiving 4V cannulas and minipumps to infuse vehicle or OT (16 nmol/day) over 29 days as previously described [28]. Daily food intake and BW were also tracked for 29 days. Animals were euthanized by rapid conscious decapitation at 9 weeks post-sham or denervation procedure. Trunk blood and tissues (IBAT, EWAT, IWAT, liver and pancreas) were collected from 4-h fasted rats and tissues were subsequently analyzed for IBAT NE

bioRxiv preprint doi: https://doi.org/10.1101/2024.09.12.612710; this version posted September 19, 2024. The copyright holder for this preprint (which was not certified by peer review) is the author/funder. This article is a US Government work. It is not subject to copyright under 17 USC 105 and is also made available for use under a CC0 license.

content to confirm success of denervation procedure relative to sham operated animals and other tissues (EWAT, IWAT, liver and pancreas).

The goal of this study was to determine if OT-elicited weight loss requires intact SNS outflow to IBAT. By design, DIO rats were obese as determined by both BW (790.7 ± 23.8 g) and adiposity (281.9 ± 16.3 g fat mass; 35.3 ± 0.1.3% adiposity) after maintenance on the HFD for 8.75 months prior to sham/denervation procedures. IBAT NE content was reduced in denervated rats by 84.8±4.3% in denervated rats relative to sham-operated control rats [(F(1,16) = 128.544, *P*=0.000)]. In contrast, NE content was unchanged in IWAT, EWAT or pancreas in denervated rats relative to sham rats (*P*=NS). In contrast, there was a reduction of NE content in liver in denervated rats relative to sham rats [(F(1,16) = 4.654, *P*=0.047)]. There was no significant difference in BW between sham and denervation groups at the end of the study (*P*=NS; data not shown).

In sham-operated rats, as expected, 4V vehicle resulted in 4.3 ± 1.0% weight gain relative to vehicle pre-treatment [(F(1,4) = 15.268, *P*=0.017]. In contrast, 4V OT reduced BW by 4.2 ± 1.2% relative to OT pre-treatment [(F(1,4) = 10.931, *P*=0.030] (**Supplemental Figure 3A**) and it also reduced weight gain throughout the 29-day infusion period (**Supplemental Figure 3B**). OT treatment reduced weight gain on days 4-29 (*P*<0.05) and tended to reduce weight gain on day 3 (*P*=0.068). 4V OT reduced relative fat mass (pre- vs post-intervention; **Supplemental Figure 3C**) (*P*<0.05) and also produced a reduction in the relative lean body mass (pre- vs post-intervention; *P*<0.05) without impacting total fat mass or lean body mass (*P*=NS). These effects that were mediated, at least in part, by a modest reduction of energy intake that persisted throughout the first two weeks of the treatment period (**Supplemental Figure 3D**; *P*<0.05). 4V OT also tended to reduce energy intake during weeks 3 and 4 of the treatment period (**Supplemental Figure 3D**; 0.05<*P*<0.1).

In denervated rats, as expected, 4V vehicle resulted in 4.7 ± 0.8% weight gain relative to vehicle pre-treatment [(F(1,3) = 44.734, *P*=0.007] (**Supplemental Figure 3A**). In contrast, 4V OT failed to reduce BW relative to OT pre-treatment but it reduced weight gain

bioRxiv preprint doi: https://doi.org/10.1101/2024.09.12.612710; this version posted September 19, 2024. The copyright holder for this preprint (which was not certified by peer review) is the author/funder. This article is a US Government work. It is not subject to copyright under 17 USC 105 and is also made available for use under a CC0 license.

throughout the 29-day infusion period (**Supplemental Figure 3B**). OT treatment reduced weight gain on days 4-29 (*P*<0.05) and it tended to reduce weight gain on day 3 (*P*=0.05). 4V OT reduced relative fat mass (pre- vs post-intervention; **Supplemental Figure 3C**) (*P*<0.05) without effecting total fat mass or lean body mass (*P*=NS). These effects that were mediated, at least in part, by a modest reduction of energy intake that was evident during weeks 1 and 2 of the treatment period (**Supplemental Figure 3D**; *P*<0.05). 4V OT also tended to reduce energy intake during week 3 of the treatment period (**Supplemental Figure 3D**; *P*=0.103).

Overall, these findings demonstrate that sympathetic innervation of IBAT is not a predominant mediator of 4V OT-elicited reductions of BW and adiposity in male DIO rats.

bioRxiv preprint doi: https://doi.org/10.1101/2024.09.12.612710; this version posted September 19, 2024. The copyright holder for this preprint (which was not certified by peer review) is the author/funder. This article is a US Government work. It is not subject to copyright under 17 USC 105 and is also made available for use under a CC0 license.

Figure legend

Supplemental Figure 1A–D: Effect of 4V OT on IBAT temperature (T_{IBAT}) in lean rats. Rats were fed *ad libitum* and maintained on low fat chow diet (13% kcal from fat) (N=6/group) for approximately 1 week prior to underdoing SNS denervation procedures and implantation of temperature transponders underneath the left IBAT depot. Rats subsequently received 4V cannulations. Rats were allowed to recover for at least 2 weeks during which time they were adapted to a daily 4-h fast, handling and mock injections. Rats subsequently received injections of 4V OT (1 or 5 μg/μL) or vehicle where each animal received each treatment at least 48-h intervals. *A*, Effect of 4V OT on T_{IBAT} in IBAT denervated lean rats; *B*, Effect of 4V OT on change in T_{IBAT} relative to baseline T_{IBAT} (delta T_{IBAT}) in IBAT denervated lean rats. Data are expressed as mean ± SEM. **P*<0.05, †0.05<*P*<0.1 4V OT vs. vehicle.

Supplemental Figure 2A–B: Effect of β3-AR antagonist, SR59230A, on the ability of the β3-AR agonist, CL 316243, to increase T_{IBAT} in DIO rats. Rats were maintained on HFD (60% kcal from fat; N=17/group) for approximately 4.25 months prior to being implanted with temperature transponders underneath IBAT. Animals were subsequently adapted to a 4-h fast prior to receiving acute IP injections of the β3-AR agonist, SR59230A or vehicle approximately 20 minutes prior to IP injections of the β3-AR agonist, CL 316243. *A*, Effect of the β3-AR antagonist (SR-59230A) pre-treatment on the ability of the β3-AR agonist, CL 316243, to stimulate T_{IBAT} at 0.5-h post-injection; *B*, Effect of the β3-AR antagonist (SR-59230A) pre-treatment on the ability of the β3-AR agonist, CL 316243, to stimulate T_{IBAT} at 0.75-h post-injection. Data are expressed as mean ± SEM. *P*<0.05, †0.05<*P*<0.1 OT vs. vehicle.

Supplemental Figure 3A–D: Effect of chronic 4V OT infusions (16 nmol/day) on BW, adiposity and energy intake post-sham or IBAT denervation in male DIO rats with more pronounced diet-induced obesity. *A*, Rats were maintained on HFD (60% kcal from fat; N=4-5/group) for approximately 8.75 months prior to undergoing a sham or bilateral surgical IBAT denervation. Rats were subsequently implanted with 4V cannulas

bioRxiv preprint doi: https://doi.org/10.1101/2024.09.12.612710; this version posted September 19, 2024. The copyright holder for this preprint (which was not certified by peer review) is the author/funder. This article is a US Government work. It is not subject to copyright under 17 USC 105 and is also made available for use under a CC0 license.

and allowed to recover for 2 weeks prior to being implanted with subcutaneous minipumps that were subsequently attached to the 4V cannula. *A*, Effect of chronic 4V OT or vehicle on BW in sham operated or IBAT denervated DIO rats; *B*, Effect of chronic 4V OT or vehicle on BW change in sham operated or IBAT denervated DIO rats; *C*, Effect of chronic 4V OT or vehicle on adiposity in sham operated or IBAT denervated DIO rats; *D*, Effect of chronic 4V OT or vehicle on adiposity in sham operated or IBAT denervated DIO rats. Data are expressed as mean ± SEM. *$P<0.05$, †$0.05<P<0.1$ OT vs. vehicle.

bioRxiv preprint doi: https://doi.org/10.1101/2024.09.12.612710; this version posted September 19, 2024. The copyright holder for this preprint (which was not certified by peer review) is the author/funder. This article is a US Government work. It is not subject to copyright under 17 USC 105 and is also made available for use under a CC0 license.

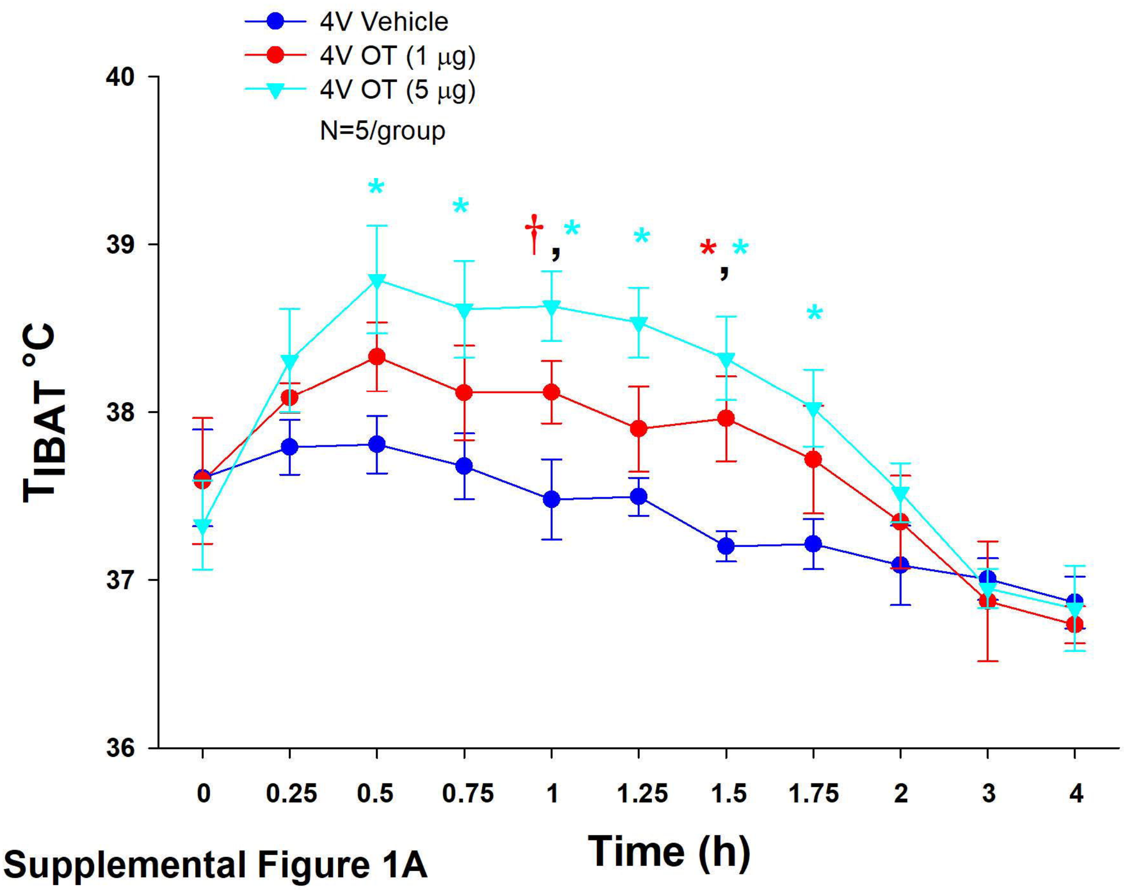

Supplemental Figure 1A

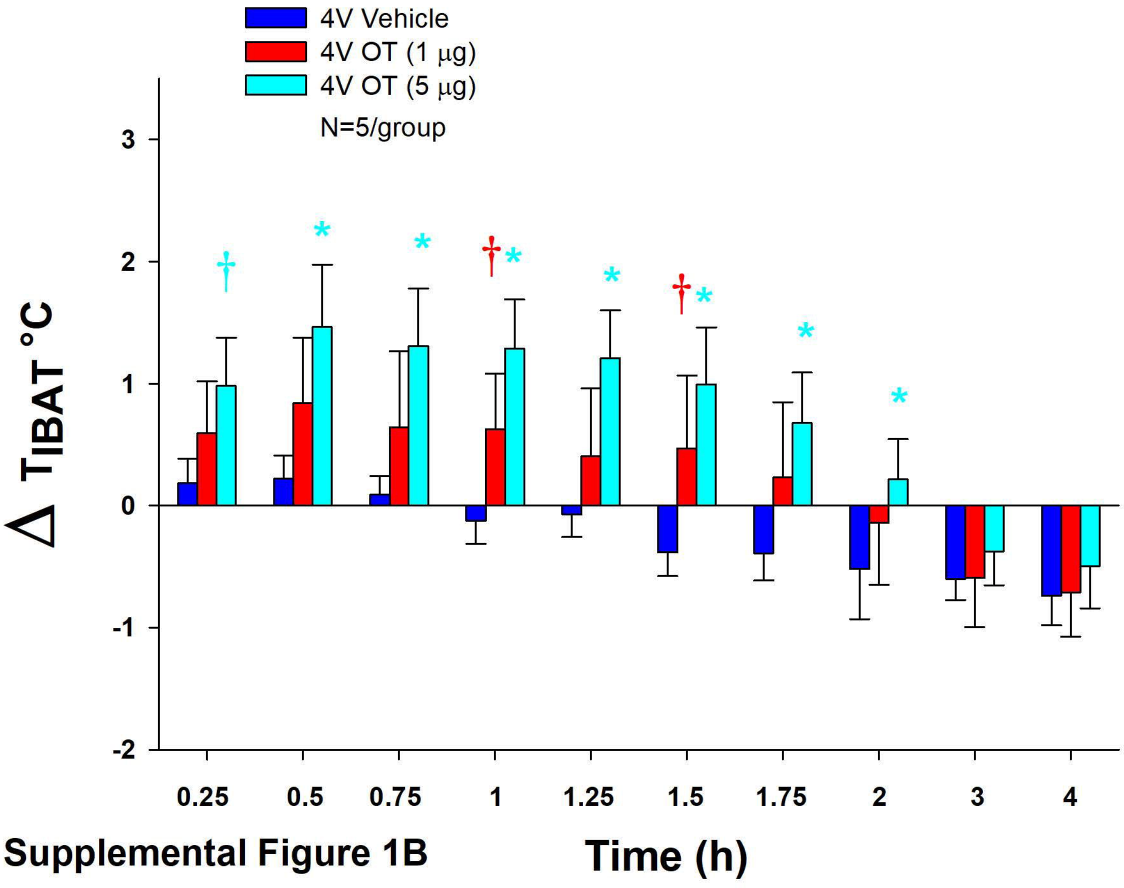

Supplemental Figure 1B

bioRxiv preprint doi: https://doi.org/10.1101/2024.09.12.612710; this version posted September 19, 2024. The copyright holder for this preprint (which was not certified by peer review) is the author/funder. This article is a US Government work. It is not subject to copyright under 17 USC 105 and is also made available for use under a CC0 license.

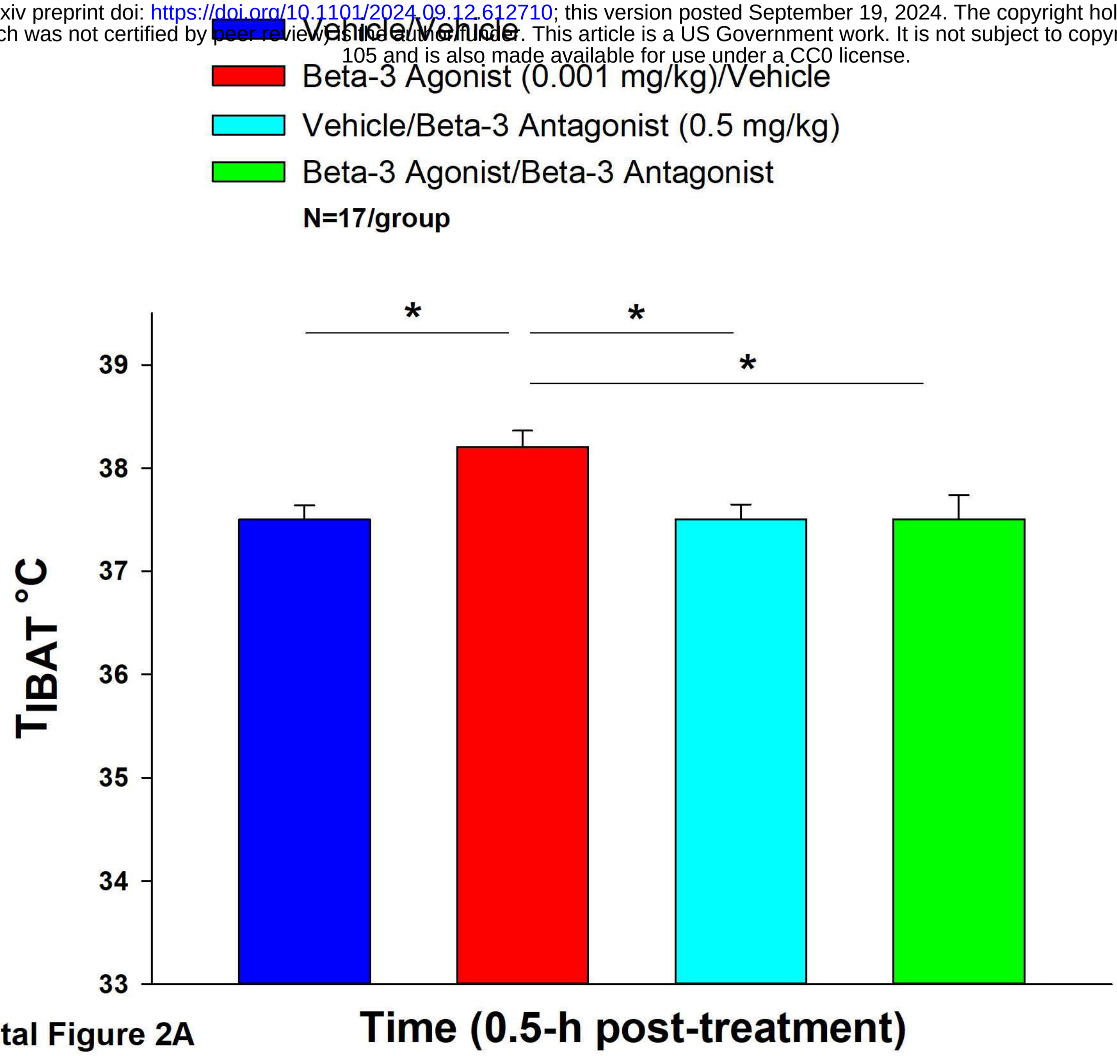

Supplemental Figure 2A

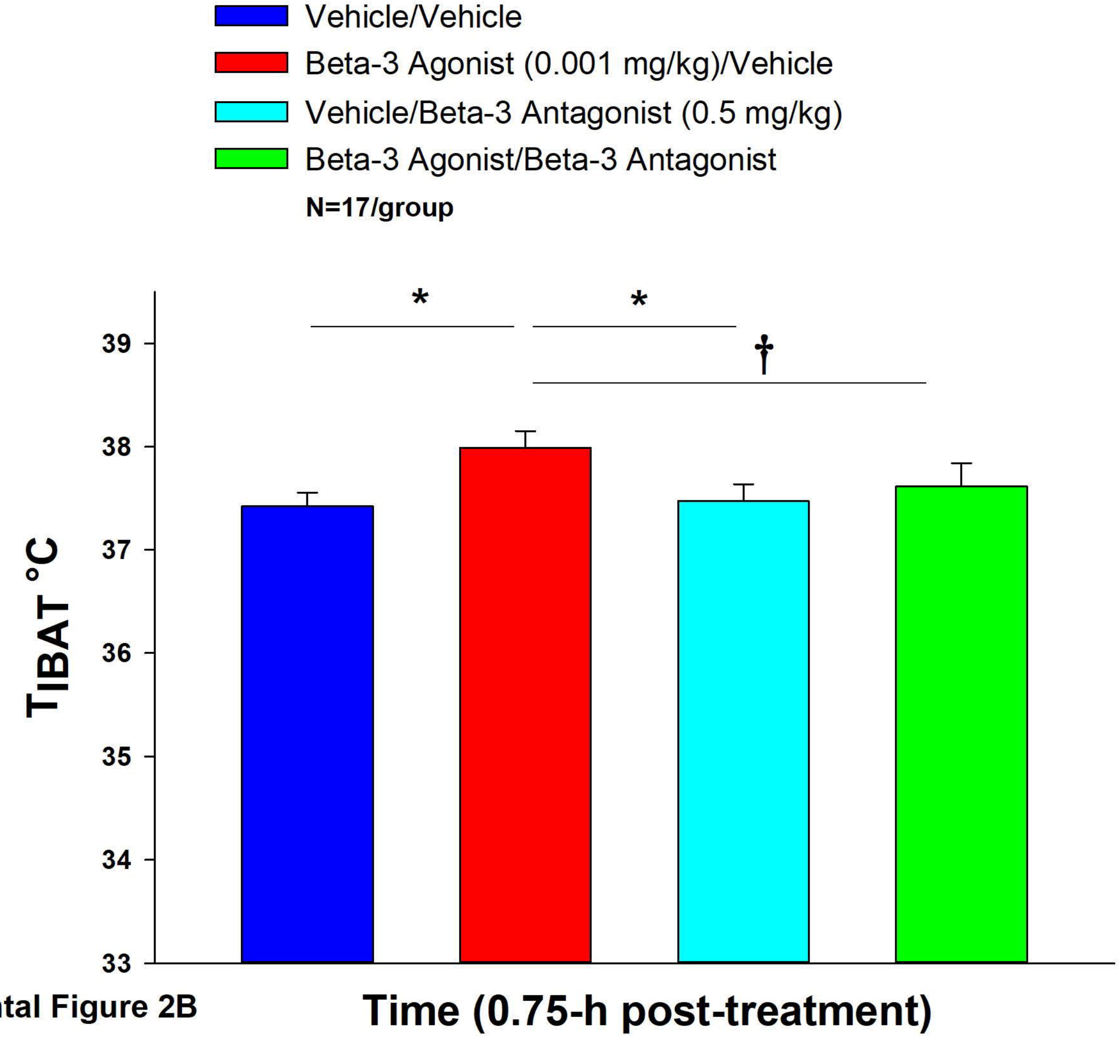

Supplemental Figure 2B

bioRxiv preprint doi: https://doi.org/10.1101/2024.09.12.612710; this version posted September 19, 2024. The copyright holder for this preprint (which was not certified by peer review) is the author/funder. This article is a US Government work. It is not subject to copyright under 17 USC 105 and is also made available for use under a CC0 license.

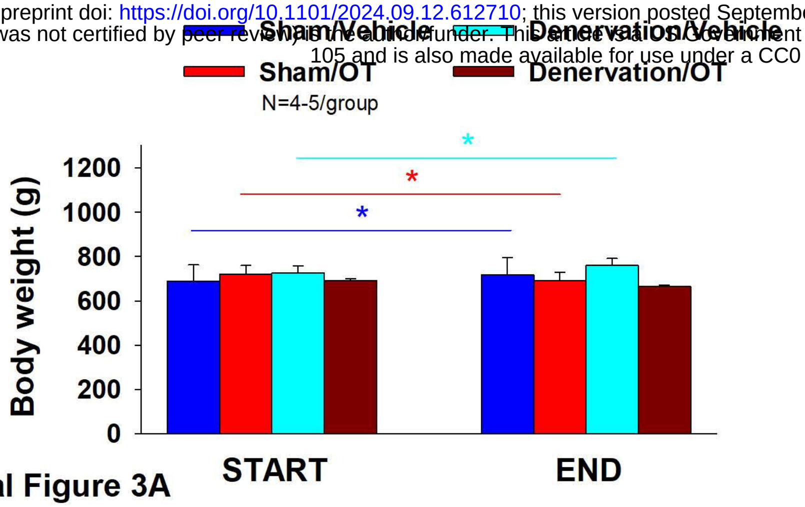

Supplemental Figure 3A

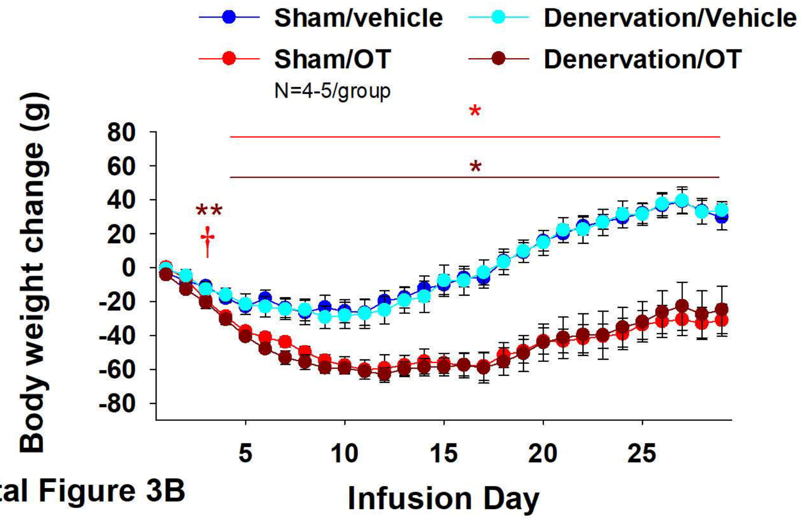

Supplemental Figure 3B

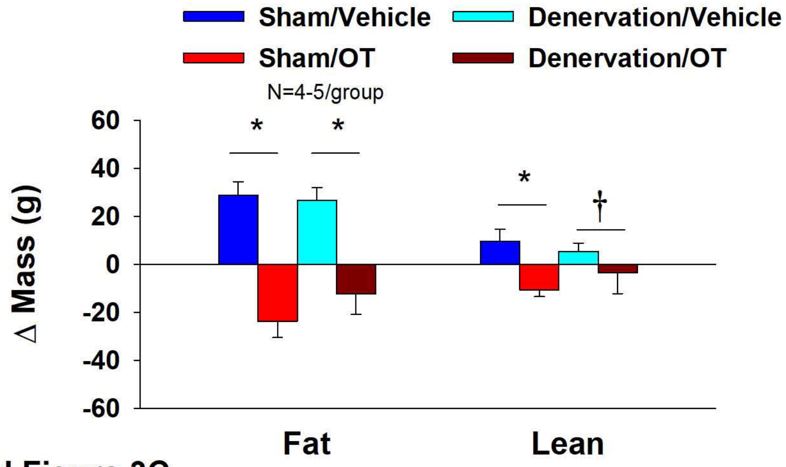

Supplemental Figure 3C

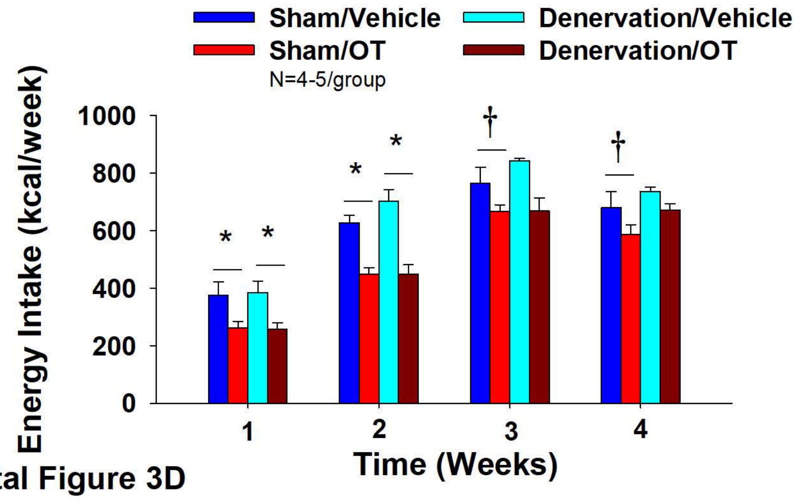

Supplemental Figure 3D
